# Preparation, Stability and In Vitro Antineoplastic Function of Lecithin–Chitosan–Polyethylene Glycol Nanoparticles Loaded with Bioactive Peptides Derived from Phycocyanin

**DOI:** 10.3390/foods14203487

**Published:** 2025-10-13

**Authors:** Haozhe Cheng, Binyang Jia, Xinran Li, Yali Li, Boxiong Wu, Qi Yang, Chengtao Wang, Baoguo Sun, Shuai Hao

**Affiliations:** Key Laboratory of Geriatric Nutrition and Health (Beijing Technology and Business University), Ministry of Education, Beijing Engineering and Technology Research Center of Food Additives, Beijing Technology and Business University, Beijing 100048, China

**Keywords:** phycocyanin, bioactive peptides, liposome, LEC–CS–PEG, cell function

## Abstract

Phycocyanin (PC) is a type of alga-derived protein which exerts the role of light harvesting in *Spirulina* and *Cyanophyta* cells. Studies have widely proved that phycocyanin exhibits antineoplastic functions, while investigations on its bioactive peptides remain poorly documented. In previous work, three phycocyanin-derived peptides (PCPs: PCP1-3), which exerted anticancer effects in non-small cell lung cancer (NSCLC) cells, were successfully identified. In consideration of the in vitro instability of bioactive peptides, this study firstly investigated the stabilization and function of phycocyanin-derived peptides loaded by nanoparticles (NPs). Herein, Lipid-core NPs (PCPs@LEC–CS–PEG, diameter less than 100 nm) were prepared by interfacial deposition of a polymer using lecithin (LEC, liposome core shell), chitosan (CS, coating material) and polyethylene glycol (PEG, stabilizer). The results indicate that the embedding of LEC liposomes could significantly increase the stability of PCPs through promoting their resistance to high temperature (68.256 ± 3.26%), pH (60.17 ± 3.67%) and protease. Moreover, the modification of NPs by PEG and CS could enhance the protective effects on PCPs. Furthermore, in vitro phenotypic experiments confirmed that the inclusion of PCPs@PEG-CS–LEC NPs also significantly increased the inhibitory activities of PCPs against multiple NSCLC cells including A549, H1299 and LTEP-a2 cells, compared with non-embedded PCPs. The results of this work could lay a theoretical foundation for the further development and utilization of peptides derived from phycocyanin, and also for the investigation of the antineoplastic effects of bioactive peptides.

## 1. Introduction

Phycocyanin (PC) is a well-known protein-based functional food additive derived from the photosynthetic pigment protein in *Spirulina* and *Cyanophyta* cells [1]. The biological role of phycocyanin in the antitumor field has become widely accepted over the past few decades [2]. It has been demonstrated that phycocyanin exhibits an important regulatory role in inhibiting the activity of non-small cell lung cancer (NSCLC), one of the most common cancers with the highest mortality and second-highest morbidity worldwide [3,4,5]. As a class of biological macromolecules, the investigation of specific bioactive components (peptides) of phycocyanin has become an emerging discovery direction. For instance, Liu et al. optimized the enzymatic hydrolysis process, and discovered phycocyanin-derived peptides (PCPs) with anti-pulmonary fibrosis activities [6]. Similar results were obtained by Li et al., who found PP20, a phycocyanin-derived peptide that also exhibited a protective function against pulmonary fibrosis [7]. Strikingly, three peptides (PCP1: AGDASVLEDR, PCP2: ADSLLSGLR and PCP3: MFDAFTK) derived from phycocyanin with NSCLC cell inhibition effects were identified in our previous work [8]. The in vitro phenotypic experiments proved that the three peptides had significant inhibitory effects on NSCLC cell activities, which could provide theoretical support for the potential treatment of NSCLC and the application of phycocyanin-derived functional peptides.

Despite their good water solubility, poor in vitro/in vivo stability, low bioavailability, and early clearance by the strong acidic stomach environment limit the serum half-life and the functions of PCPs [9,10], which is an important barrier that a peptide encounters during its utilization process. Peptides need to resist the action of digestive enzymes during their transit through the gastrointestinal tract and cross the intestinal epithelial barrier to reach intact the target organs where peptides can exert their health-promoting effects [11]. In order to protect food-derived peptides from the action of digestive enzymes, improve their intestinal permeability, or retain/potentiate their biological activity, different modifications of the peptide’s structure have been suggested including changes in N- and C-amino acid terminals, cylization, or side chain modifications of peptides [12,13,14]. However, traditional chemical modifications (N- and C-terminal alteration or cyclization) often require complex, multi-step synthesis and may inadvertently compromise the peptide’s bioactivity [15]. Encapsulating a peptide in nanoparticles can prevent degradation and increase stability, enabling its use in therapeutic doses, which is a highly effective and low-cost method for protecting bioactive peptides [16].

Nanoparticles (NPs) have attracted significant attention due to their unique physical and chemical properties that allow them to encapsulate, protect and control the targeted release of molecules in tissues [17]. Chitosan (CS) is a natural biodegradable polymer that is routinely used in the preparation of polymeric NPs for the protection and delivery of functional molecules [18]. The chitosan on the surface of NPs has been known to impart important mucoadhesive properties, thus increasing the permeation of NPs across cellular membranes [19]. On the other hand, chitosan–lecithin NPs were successfully applied for the stabilization, protection and functional study of many drugs or natural molecules. An investigation reported by Dartora et al. has suggested that chitosan hydrogel containing MK2 inhibitor peptide (YARA)-loaded NPs could be a promising new formulation for the topical treatment of atopic dermatitis [20]. It has also been proved that chitosan–carboxymethyl chitosan NPs exhibited strong potential as immunological adjuvants [21].

Three phycocyanin-derived peptides (PCPs: PCP1-3) with an NSCLC cell inhibition function were identified in previous work. A significant body of study has documented bioactive peptides derived from phycocyanin [22,23]. However, their therapeutic potential is severely limited by challenges like enzymatic degradation in the gastrointestinal tract and poor membrane permeability, leading to low bioavailability. Conventional approaches have failed to adequately overcome these hurdles. Notably, advanced delivery systems, particularly nanoparticle encapsulation, have not been investigated for this specific application, which presents a major obstacle to their development. Therefore, in this work, PCP-loaded NPs were prepared using the nano precipitation method. Lipid-core NPs were prepared by interfacial deposition of a polymer using lecithin (LEC) as the polymer liposome core shell, and polyethylene glycol (PEG) as stabilizer. Chitosan was used as the coating material to obtain the positive charge for nanoparticles (PCPs@LEC–CS–PEG). The characteristics, stability and in vitro antineoplastic effects of the NPs were systematically studied.

To our knowledge, no studies have been carried out to prepare PCP-loaded chitosan lecithin NPs for stability and functional investigation. The results of this work can lay a theoretical foundation for the further development and utilization of peptides derived from phycocyanin, and also for the investigation of the antineoplastic effects of bioactive peptides.

## 2. Materials and Methods

### 2.1. Materials and Reagents

Phycocyanin-derived anticancer peptides (PCPs, >98% purity) were purchased from Sangon Biotech Co., Ltd. (Shanghai, China). Egg yolk lecithin and cholesterol were purchased from Shanghai Yuanye Biotechnology Co., Ltd. (Shanghai, China). Chitosan (MW 30,000; MW 100,000; MW 200,000) was obtained from Shanghai Yuanye Biotechnology Co., Ltd. (Shanghai, China). Polyethylene glycol (PEG-2000) was purchased from Shanghai Yuanye Biotechnology Co., Ltd. (Shanghai, China). Tween-80 was purchased from Guangdong Guanghua Sci-Tech Co., Ltd. (Guangzhou, China). The BCA protein assay kit and bicinchoninic acid (BCA) solution were purchased from Beyotime Biotechnology (Shanghai, China). The PBS buffer (0.01 mol/L, pH 7.4) was obtained from Hyclone, Cytiva (Logan, UT, USA). All other chemicals and reagents were of analytical grade.

### 2.2. The Preparation of Lecithin NPs (LEC NPs)

Egg yolk lecithin and cholesterol were dissolved in ethanol solution according to the ratio of 3:1, followed by rotation and evaporation at 30× *g*/min and 55 °C for 20 min. During the rotary evaporation process, ethanol also gradually evaporated, forming a uniform transparent lipid film. Then, 50 mL of PBS buffer (0.01 mol/L; pH 7.4) containing 0.1 g of phycocyanin peptide and Tween-80 (exhibited as a protectant) was added into a round bottom flask, followed by hydrating at 60 °C and 55× *g*/min for 1 h to obtain a crude liposome solution. The solution was sonicated at 44 °C for 30 min (ultrasonic frequency 20 kHz; power 500 W; working at an interval of 2 s) to obtain liposomes. After sonication, the liposome suspension was kept at 4 °C for 12 h to improve stability. For the preparation of a freeze-dried liposome powder, the solution was frozen in a −80 °C refrigerator for 24 h, then placed in a vacuum freeze-drying machine (LYO-1, Shanghai Tofflon Sci &Tech Co., Ltd., Shanghai, China) for 48 h in the main drying mode. The freeze-dried liposome powder was collected for subsequent research. Negative control experiments were performed using liposomes prepared with the same procedure but without the addition of phycocyanin peptide, in order to evaluate the background effects of the liposome carrier itself. All experiments were repeated in triplicate to ensure reproducibility.

### 2.3. The Encapsulation Efficiency Detection of PCPs in NPs

The peptides (PCP1-3) were screened and identified in our previous work [8]. In this study, the PCP1-3 peptides (>98% purity, 400 μg/mL) were synthesized and obtained from Sangon Biotech Co., Ltd. (Shanghai, China). The concentration of the phycocyanin peptide in the liposomes was determined using an ultrafiltration membrane-based indirect method. A total of 4 mL of prepared liposomes was added into a 5 kDa ultrafiltration tube for centrifugation at 1000× *g*/min for 15 min to collect the lower layer liquid (free phycocyanin peptide solution). The lower layer of free liposomes was diluted to 2 mL with PBS buffer, followed by mixing with BCA buffer (Shanghai Beyotime Biotechnology Co., Ltd.) at 37 °C for 30 min, and the absorbance was measured at 562 nm to calculate the encapsulation efficiency. The BCA standard curve was prepared by diluting bovine serum albumin (BSA) to different concentrations (0, 25, 125, 250, 500, 750, and 1000 μg/mL) with PBS buffer. Each concentration (200 μL) was mixed with 200 μL of BCA working solution, and incubated at 37 °C for 30 min. The absorbance was measured at 562 nm. A standard curve was drawn with protein concentration as the abscissa and absorbance value as the ordinate, and this was used to calculate the concentration of peptides in the liposomes. Negative control experiments were performed with blank liposomes (without peptides) under the same conditions to eliminate the interference of liposome components with the absorbance measurements. All experiments were repeated in triplicate for statistical reliability.

(1) $$\mathrm{Embedding}\;\mathrm{rate}\;(\%)=\frac{M(Total\;PCPs)-C(Free\;PCPs)\times V1\times (V2/V3)}{M(Total\;PCPs)}$$

M (total PCPs): mass of total peptide (mg); C (free PCPs): concentration of free peptide (mg/mL); V1: fix the free peptide to 2 mL after ultrafiltration and centrifugation; V2: total volume of liposomes; V3: the volume of solution taken from the total volume is 4 ml.

### 2.4. The Preparation of Polyethylene Glycol–Chitosan–Lecithin NPs (LEC–CS–PEG NPs)

First, 1 g of chitosan with molecular weights of MW 30,000, MW 100,000, and MW 200,000 were dissolved in 100 mL of 1% acetic acid solution, respectively, to obtain a chitosan stock solution. The three different molecular weights of chitosan stock solutions were mixed with liposomes in different ratios (5:1, 4:1, 3:1, 2:1, 1:1, 1:2, 1:3, 1:4, and 1:5), followed by stirring for 2 h to form a chitosan–algal blue peptide liposome solution. After stirring, the solution was kept at 4 °C for 12 h to allow sufficient interaction between the chitosan and liposome membranes.

**Figure 1:**
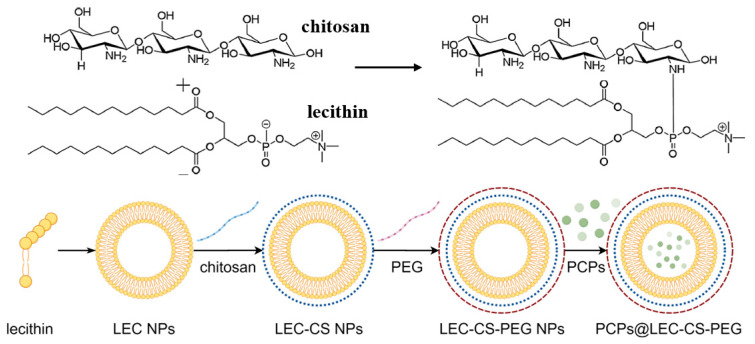
Schematic representation of chitosan–lecithin co-polymer and PCPs@LEC–CS–PEG NPs.

Then, 0.5 g of PEG-2000 was dissolved in 50 mL of ultrapure water, followed by stirring for 30 min to prepare the PEG-2000 solution. Then PEG-2000 was added into liposome buffers with different concentrations (2%, 4%, 6%, 8%, and 10%). After PEG addition, the liposome suspension was stirred for 1 h at room temperature, then stored at 4 °C overnight to enhance stability. Negative control experiments were performed by preparing liposomes without chitosan or PEG under the same conditions, in order to distinguish the effects of each modification. All experiments were performed in triplicate. A schematic representation is shown in Figure 1.

### 2.5. The Particle Size, PDI and Zeta Potential Measurements

The particle size, PDI, and zeta potential (ζ-potential) of the liposome samples were measured using a Malvern laser particle sizer (Mastersizer 3000, Spectris plc, Malvern, British). The experimental conditions were set to a temperature of 25 °C, an angle of 90°, a refractive index of 1.330, and a dispersion viscosity of 0.8872 cP. Before measurement, the samples were diluted three times with PBS (0.01 mmol/L, pH 7.4). The process was repeated three times, and the average value was taken.

### 2.6. Fourier Transform Infrared (FTIR) Spectroscopy Detection

The lyophilized liposomes were blended with dried KBr powder (Yuanye Bio-Technology Co., Ltd., Shanghai, China) at a ratio of 1:200 (m:m). The mixture was compressed into transparent and crack-free sheets on a tablet press, and the sheets were mounted on a sample holder. The scanning range of the spectrometer was 4000–400 cm^−1^. The resolution and number of scans of FTIR were 4 cm^−1^ and thirty-two, respectively.

### 2.7. The Thermal Stability Assessment of the Prepared NPs

The sample was heated in an 80 °C water bath for 1 h. Then, 4 mL of the sample was collected at 20, 40, and 60 min, respectively, for the PDI, particle size and ζ-potential measurement. The process was repeated three times.

### 2.8. Cell Viability Assays

A549, H1299 and LTEP-a2 cell lines were provided by American Type Culture Collection (ATCC). Cells were cultured in DMEM media (Invitrogen, Long Island, NY, USA) supplemented with 10% fetal bovine serum (FBS, HyClone, Logan, UT, USA), streptomycin (100 μg/mL) and penicillin (100 units/mL) in a humidified incubator with 5% CO_2_ at 37 °C. The cell state was normal and there was no contamination by mycoplasma. MTT was used for viability detection. Briefly, cells were seeded at 5000 cells in 100 μL of complete medium per well in quadruplicate in 96-well plates. After 12 h of incubation for cell attachment, cells were conducted with 400 μg/mL of PCPs, PCPs@LEC–CS and PCPs@LEC–CS–PEG, respectively. Each day, 10 μL MTT/well was added to test cells and incubated for 4 h at 37 °C. Then, a SDS–HCl solution (10% SDS, 0.01 M HCl) was added into each well and incubated for 14 h at 37 °C. The assay lasted for 4 days after treatment. The cell viability is presented as the ratio of the absorbance reading and the control cells. The absorbance was measured at 570 nm (detection wavelength) and 490 nm (reference wavelength).

### 2.9. Transmission Electron Microscope (TEM) Observation

The morphology of liposomes was observed by transmission electron microscopy. A drop of freshly prepared liposome suspension was placed on a carbon-coated copper grid, and excess liquid was removed with filter paper. The sample was negatively stained with 1% uranyl acetate solution (acetic acid–uranyl reagent) for 2 min. After staining, the grid was air-dried at room temperature and then observed under a TEM (80 kV accelerating voltage).

### 2.10. Statistical Analysis

Three independent biological replicates were employed in this study. GraphPad Prism (version 9) was used to assess significant differences (*p* < 0.05 was considered as difference, and *p* < 0.01 was considered as significant difference) using a Student’s *t*-test or an analysis of variance (ANOVA), followed by Tukey’s honest significant difference (HSD) test for multiple comparisons. The results are presented as the mean s ± SD from three independent experiments.

## 3. Results

### 3.1. Preparation of PCP-Loaded LEC–CS NPs

LEC–CS NPs loaded with PCPs (PCP1, PCP2 and PCP3) were prepared. The molecular weight of CS may have an effect on the formation and stability of LEC–CS NPs, so the particle size, ζ-potential and PDI levels were measured first. It is known that a low PDI value (0.1–0.2) and a high ζ-potential (over 20) indicate a more stable solution environment. When the ζ-potential is between 5 and 20, it suggests that the solution can maintain a certain stable state even in a short-term environment [24,25].

**Figure 2:**
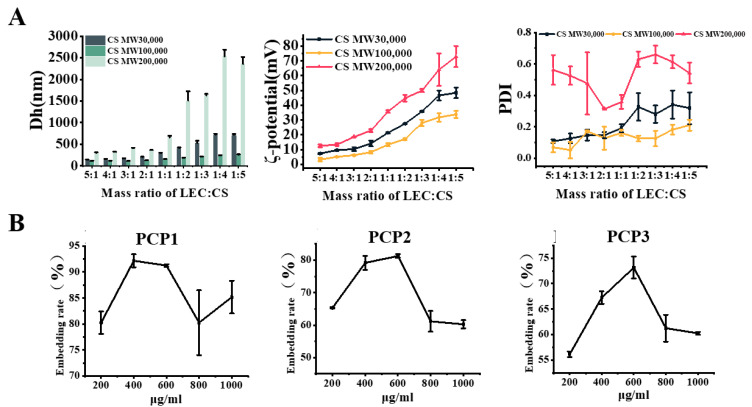
Preparation of PCPs@LEC–CS NPs. (**A**) The particle size (**left**), ζ-potential (**middle**) and PDI levels (**right**) of CS–LEC NPs. Different molecular weight of CS (MW 30,000, MW 100,000 and MW 200,000) were used in this experiment. For particle size, dark green columns indicate CS of MW 30,000, green columns indicate CS of MW 100,000, and light green columns indicate CS of MW 200,000. For ζ-potential and PDI levels, dark lines indicate CS of MW 30,000, yellow lines indicate CS of MW 100,000, and red lines indicates CS of MW 200,000. (**B**) Determination of the embedding rate of liposome nanoparticles by concentrations of PCP1, PCP2 and PCP3. Bars represent mean ± SD.

As shown in Figure 2A, in general, with the increasing addition of CS, the particle size and PDI showed a trend of increasing, indicating that the liposomes become more and more unstable in solution. In particular, CS with a large molecular weight (MW 200,000) could not form stable NPs with LEC. When the ratio of LEC to CS was 1:4, the particle size and PDI value of the liposome reached 2526 ± 31 nm and 0.61 ± 0.23, respectively, suggesting that the flocculation between CS and LEC increased rapidly. Likewise, CS with a molecular weight of 30,000 caused similar results (Figure 2A). However, it is worth mentioning that the particle size and PDI value of LEC–CS NPs were sustained at a relatively stable level under different addition amounts of CS with a molecular weight of 100,000. When the ratio of LEC to CS was 3:1, the NPs showed the minimum particle size (109 ± 12 nm). The PDI and ζ-potentials of liposomes were 0.17 ± 0.05 and 6.19 ± 1.23 mV, respectively, revealing that the LEC–CS binding was tight and stable (Figure 2A). Taken together, a 3:1 ratio of LEC to CS (MW 100,000) was selected for subsequent experiments.

Under the above optimal conditions for LEC–CS NPs, the embedding rate of peptides by liposomes was further determined. As shown in Figure 2B, the concentration and type of polypeptides could affect the embedding efficiency of liposome. For PCP1, the optimal embedding concentration is 400 μg/mL, while 600 μg/mL is the optimal embedding dose for PCP2 and PCP3 liposomes. Taken together, the above results show the optimal conditions for LEC–CS NPs loaded with PCPs (PCPs@LEC–CS), which has laid a foundation for our further study.

### 3.2. Effects of PEG-2000 on the Stabilities of PCPs@LEC–CS

**Figure 3:**
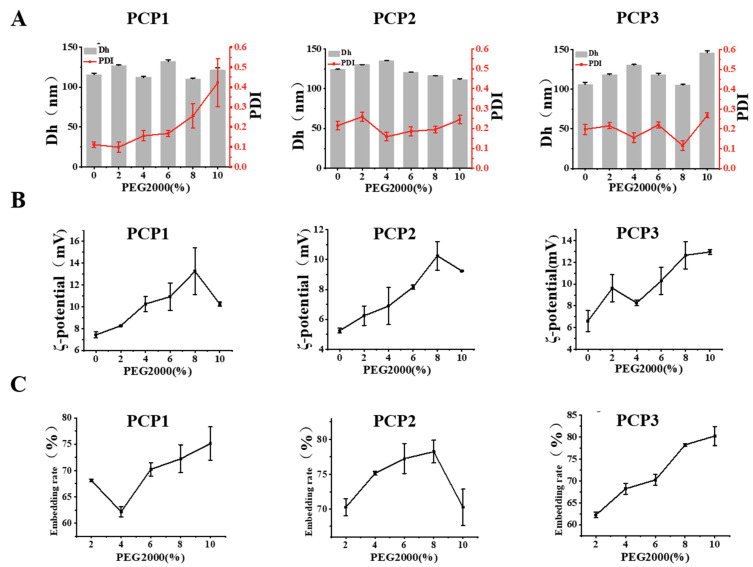
The effects of PEG-2000 on the stabilities of LEC–CS NPs loaded with peptides. (**A**) Effects of PEG-2000 on the particle size (shown as grey columns) and PDI values (shown as red lines) of LEC–CS NPs. (**B**) Effects of PEG-2000 on ζ-potential of LEC–CS NPs. (**C**) Effects of PEG-2000 on the embedding efficiency of peptides by LEC–CS NPs. Bars represent mean ± SD.

It has been reported that polymer compounds may have a certain enhancement effect on the stability of liposome NPs [26]. In this work, PEG-2000 was used to explore its effect on the stability of PCPs@LEC–CS NPs. As shown in Figure 3A, with the increase of PEG concentration (0, 2, 4, 6, 8, and 10%), although the PDI value of PCP1@LEC–CS liposomes showed a weak dose-dependent increase effect, the particle size and PDI (lower than 0.4) of three liposomes remained relatively stable, indicating that PEG-2000 is able to stabilize the structure of PCPs@LEC–CS NPs. Meanwhile, the effects of PEG on the embedding rate and ζ-potential of NPs were also investigated. Interestingly, the embedding efficiency of NPs on peptides and ζ-potential were both affected by PEG concentration. It is worth mentioning that when the dose of PEG-2000 was 8%, the embedding effects of NPs on the three peptides reached over 70% (Figure 3C), as well as exhibiting high ζ-potential (Figure 3B). Taken together, the above results suggested that PEG could be acting as a stabilizer for PCPs@LEC–CS NPs, and 8% of PEG concentration was selected for the subsequent experiments.

### 3.3. Transmission Electron Microscope Observation of the Micromorphology of NPs

**Figure 4:**
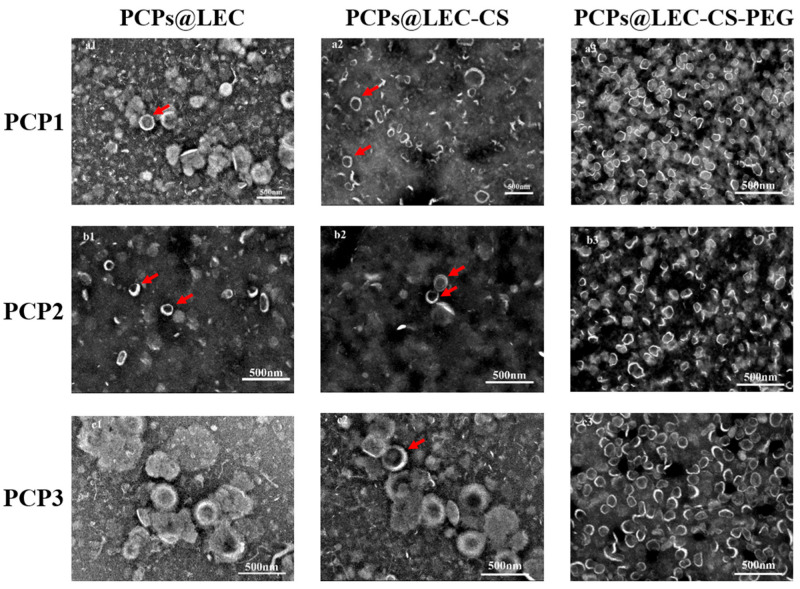
Transmission electron microscope observation of micromorphology of NPs including (**a1**) PCP1@LEC, (**a2**) PCP1@LEC–CS, (**a3**) PCP1@LEC–CS–PEG, (**b1**) PCP2@LEC, (**b2**) PCP2@ LEC–CS (**b3**) PCP2@LEC–CS–PEG, (**c1**) PCP3@LEC, (**c2**) PCP3@LEC–CS, and (**c3**) PCP3@LEC–CS–PEG. The red arrow indicates the image of the liposome under an electron microscope.

The micromorphology of NPs loaded with PCPs was examined through TEM. As shown in Figure 4, LEC could only form a small number of NPs loaded with PCPs, and the surface of the liposome was rough (marked by red arrows in the first line). After CS addition, compared with LEC groups, although the surface of the liposomes became smoother, the formation rate of NPs was still low (marked by red arrows in the second line). Notably, it was observed that a large number of smooth and stable liposome particles were formed after being stabilized by PEG. Meanwhile, the diameters of the liposomes were also relatively uniform (less than 100 nm). Taken together, the micromorphology results further verified that LEC–CS–PEG could form stable NPs loaded with PCPs.

### 3.4. Fourier Transform Infrared Spectroscopy Detection of PCPs@LEC–CS–PEG

**Figure 5:**
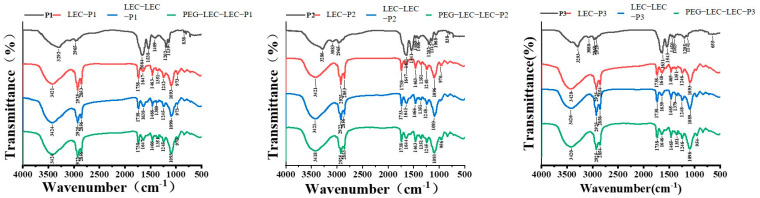
FTIR analysis of different samples including PCPs (black curves), PCPs@LEC (red curves), PCPs@LEC–CS (blue curves), and PCPs@LEC–CS–PEG (green curves).

Figure 5 illustrates the FTIR spectrum of four different types of samples including PCPs (black curves), PCPs@LEC (red curves), PCPs@LEC–CS (blue curves), and PCPs@LEC–CS–PEG (green curves). According to the FTIR spectra, the pattern of bands in PCPs are different from that in liposomes. Six bands were ascribed to active groups in phycocyanin-derived peptides (3290, 3000, 1664, 1538, 1406, and 838 cm^−1^). The peak at 3290 cm^−1^ corresponds to the amide I band derived from the N–H stretching vibration, while the absorption band at 3000 cm^−1^ originates from the O–H stretching vibration. The bands at 1664, 1538, and 1406 cm^−1^ are attributed to amide I (C=O stretching vibration) together with random coil structures, and amide III (C–O stretching vibration). The band at 838 cm^−1^ arises from the combined C=O stretching and N–H bending vibration, while in liposome NPs (PCPs@LEC, PCPs@LEC–CS, and PCPs@LEC–CS–PEG), the characteristic peaks mainly occur between 3421 to 973 cm^−1^. The O-H hydrogen bond occurred at approximately 3421 cm^−1^. The bands at 1738 and 1647 cm^−1^ correspond to the C=O stretching vibration of ester bonds in the lipid acyl chain and phospholipid head groups. In particular, a new characteristic peak created by the tension vibration of PO^2-^ has occurred at 1242 cm^−1^. These findings have suggested that the existence of an LEC–peptide complex might help to strengthen the interaction between molecules [27,28], thus protecting the stability of PCPs. Moreover, the amide I band (~1664 cm^−1^, mainly C=O stretching vibration) and the amide II band (~1538 cm^−1^, mainly N–H bending vibration coupled with C–N stretching) showed distinct changes after encapsulation into liposomes. These spectral shifts demonstrate that the secondary structure of the peptides was altered by hydrogen bonding and electrostatic interactions with the lipid bilayer. In particular, the decrease in amide I intensity and the slight shift of amide II suggest that α-helix and random coil structures of the peptides were partially reorganized when interacting with lecithin, chitosan, and PEG layers.

### 3.5. The Thermal, pH and Storage Stability Analysis of PCPs@LEC–CS–PEG NPs

The above results have illustrated that CS and PEG could promote the stability and embedding efficiency of NPs loaded with PCPs. In this work, the thermal (Table S1), pH (Table S2) and storage (Table S3) stabilities of NPs have been investigated further. As shown in Table S1, compared with control groups (68.52 ± 0.052), the particle size of PCP1@LEC was 63.256 ± 1.254 after 60 min of heating at 80 °C. Analogously, the PDI value had no obvious difference between the heating (0.256 ± 0.0126) and control groups (0.2155 ± 0.021) of PCP1@LEC, revealing that high temperature had no significant effects on the particle size and PDI of PCP1@LEC NPs. Meanwhile, the embedding rate was significantly reduced by 15% (31.166% ± 2.655%) after treatment for 60 min at 80 °C, as compared with control groups (46.256% ± 0.256%), which indicated that high-temperature heating would reduce the stability of LEC, and then affect its embedding efficiency on peptide. Nevertheless, the particle size, PDI and embedding rate were relative stable in LEC–CS and LEC–CS–PEG NPs after high-temperature treatment. In terms of the embedding rate, compared with LEC liposomes, CS (53.262% ± 4.256%) and PEG (68.256% ± 3.26%) modifications resulted in loss of only 6% of peptides after heating treatment. Similar results were also found in PCP2@LEC andPCP3@LEC NPs.

With regard to pH stabilities, generally, the embedding rate of PCPs gradually decreased as pH increased. However, the LEC–CS–PEG NPs exerted a higher embedding ratio in a particular pH level, compared with LEC and LEC–CS NPs (Table S2), revealing that PCPs@LEC–CS–PEG NPs were resistant to acid and alkali. This might be due to the fact that high molecular weight PEG, through water transport interactions and hydrogen bonds, could effectively penetrate the shell of the liposome and exert a stabilizing effect on it, effectively resisting damage to the liposome particles under extreme conditions [29].

Furthermore, the storage stabilities of the liposomes were measured at 4 °C for three weeks (21 days). As shown in Table S3, although the particle sizes of all three NPs grew with the increase of time, the growing rate of the particle size of PCPs@LEC–CS–PEG (34.28% ± 3.26%) was significantly lower than that of PCPs@LEC (60.17% ± 3.67%) NPs. Taken together, these results have indicated that CS and PEG could effectively improve the thermal, pH and storage stabilities of liposomes, thereby reducing the leakage of phycocyanin-derived peptides in adverse environments.

### 3.6. Detection of the Release Rate of PCPs from NPs in Simulated Gastric Digestion In Vitro

**Figure 6:**
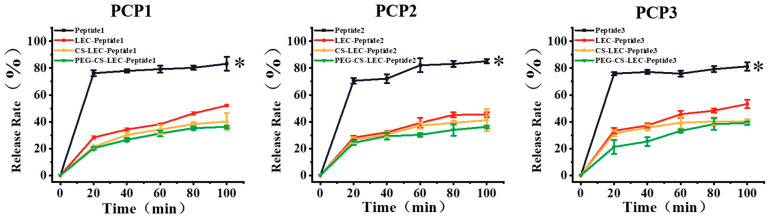
The in vitro digestive stabilities of PCPs and NPs loaded with PCPs. Black curves represent the degradation rate of PCPs after in vitro digestion by pepsin for 100 min. Blue curves represent the degradation rate of PCPs@LEC NPs after in vitro digestion by pepsin for 100 min. Yellow curves represent the degradation rate of PCPs@LEC–CS NPs after in vitro digestion by pepsin for 100 min. Green curves represent the degradation rate of PCPs@LEC–CS–PEG NPs after in vitro digestion by pepsin for 100 min. * represents *p* < 0.05. Bars represent mean ± SD.

In vitro digestion could reflect the protective ability of liposomes on peptides. In this work, the in vitro simulated gastric digestion measurements of PCPs and NPs loaded with PCPs were further employed. As shown in Figure 6, the degradation rates of PCP1 (78.82% ± 3.46%), PCP2 (71.65% ± 2.99%) and PCP3 (77.24% ± 1.72%) alone showed a significant increase after only 20 min of in vitro digestion, indicating that PCP segments are vulnerable to pepsin destruction without liposomal protection. In contrast, PCPs coated with LEC showed strong resistance to pepsin, with a degradation rate of less than 30% after 20 min of digestion (PCP1:19.19% ± 1.23%, PCP2: 22.48% ± 1.77%, and PCP3: 20.05% ± 7.45%, respectively). Particularly, LEC–CS–PEG NPs showed the strongest protective effects on three peptides, with degradation rates of 36.25% (PCP1), 36.256% (PCP2) and 39.11% (PCP3), respectively, after 100 min of digestion in vitro. Taken together, the above results suggest that the tight structure of liposomes could prevent leakage of PCPs to other mediums, which effectively slows down the release of peptides. LEC–CS–PEG NPs have a sustained-release effect of PCPs due to the protective layer on the surface of liposomes formed by CS and PEG-2000 coating.

### 3.7. The In Vitro Effects of PCPs@LEC–CS–PEG NPs on Growth of NSCLC Cells

**Figure 7:**
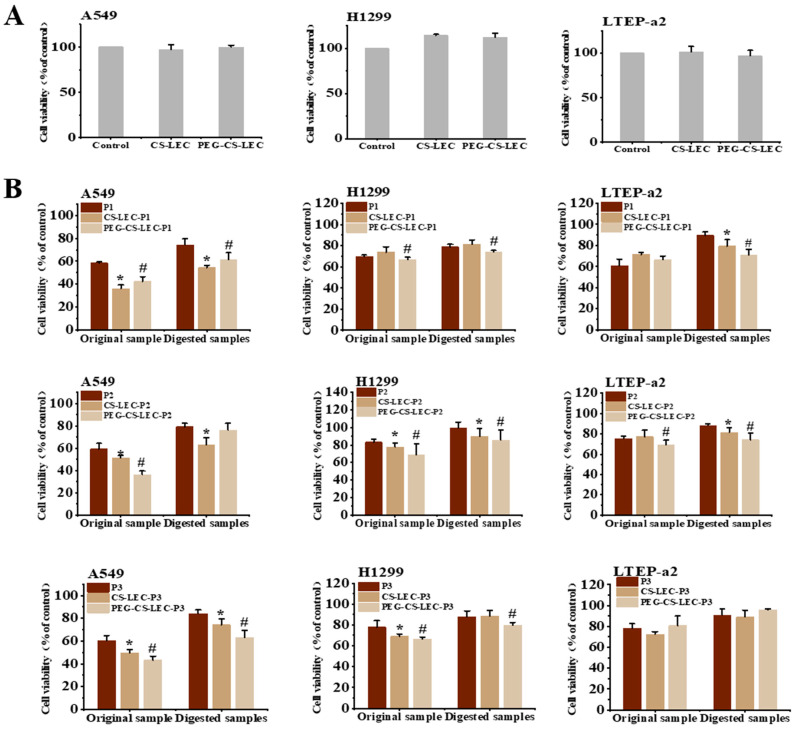
Cell viability analysis of PCPs@LEC–CS–PEG NPs on NSCLC cell lines including A549, H1299 and LTEP-a2 cells. (**A**) The cytotoxicity detection of LEC–CS and LEC–CS–PEG NPs on A549, H1299 and LTEP-a2 cells. (**B**) Cell viability analysis of PCPs (red columns), PCPs@LEC–CS (yellow columns) and PCPs@LEC–CS–PEG (light yellow columns) on NSCLC cells before and after pepsin digestion, respectively. * represents significance between PCPs@LEC–CS and PCPs groups (*p* < 0.05), # represents significance between PCPs@LEC–CS–PEG and PCPs groups (*p* < 0.05). P1–P3 represent PCP1–PCP3. Bars represent mean ± SD.

The above results have revealed that PEG and CS modifications could significantly enhance the stability and protective effect of LEC NPs on PCPs. Herein, the in vitro antineoplastic function of PCPs and NPs on NSCLC cells were further investigated. Firstly, the cytotoxicity of NPs was measured through cell viability assays. It was shown that compared with the control groups, LEC–CS and LEC–CS–PEG exposure did not significantly reduce the survival rate of the three types of NSCLC cells (Figure 7A), indicating that liposome NPs have no cytotoxic effects on cells. Subsequently, the effects of PCPs and its liposomes, before and after digestion, on cell survival were studied. As shown in Figure 7B, compared with the cells treated by PCPs alone, except for LTEP-a2 cells, the embedding of liposomes was able to significantly enhance the inhibitory effects of PCPs on A549 and H1299 cells, of which, the simultaneous modification of PEG and CS exhibited a more obvious suppression function than that of CS alone. This might be due to the fact that the embedding of LEC–CS–PEG has a better protective effect on the integrity of PCPs, which makes it more stable in exerting the antineoplastic effect in vitro. It is worth mentioning that the survival rate of NSCLC cells treated by post-digestive PCPs and their NPs was significantly increased compared with those of the undigested groups, revealing that the digestion of protease has a certain antagonistic effect on the biological activity of PCPs. Interestingly, the embedding of PCPs by LEC, especially the PEG and CS modified liposomes, exhibited better resistance to the digestion of protease, resulting in a higher antineoplastic function than PCPs alone. In short, these results have indicated that the inclusion of PCPs@LEC–CS–PEG can enhance the resistance of peptides to proteases, thereby increasing the stability of PCPs in vitro. The results could also lay a certain foundation for improving the utilization rate of functional peptides.

### 3.8. The In Vitro Effects of PCPs@LEC–CS–PEG NPs on the Migration of NSCLC Cells

**Figure 8:**
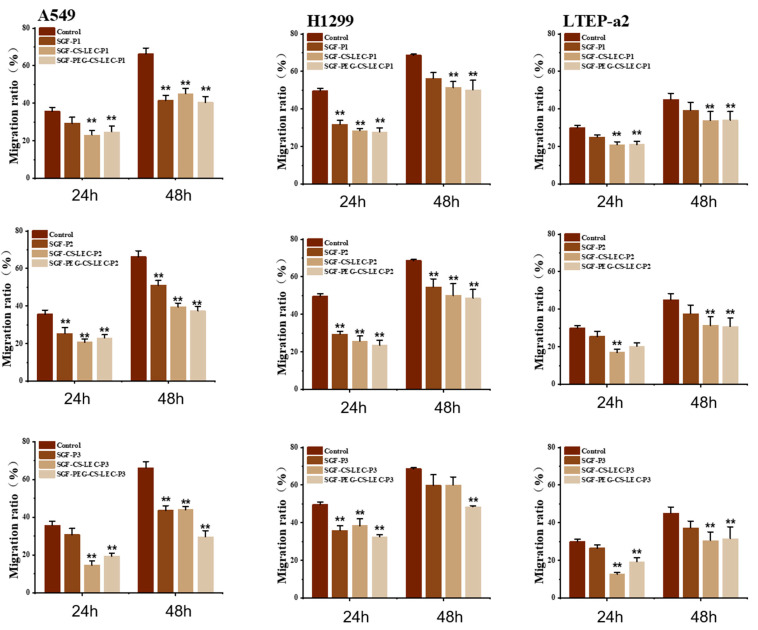
Cell migration analysis of digestive PCPs@LEC–CS–PEG NPs on NSCLC cell lines including A549, H1299 and LTEP-a2 cells. Red columns indicate control groups, orange columns indicate SGF peptide groups, yellow columns indicate SGF-PCPs@LEC–CS groups, and light yellow columns indicate SGF-PCPs@LEC–CS–PEG groups. **, *p* < 0.01. SGF, Simulated gastric fluid digestion. P1–P3 represent PCP1–PCP3. Bars represent mean ± SD.

The above results have suggested that CS and PEG protection could not only enhance the anti-digestion ability of PCPs@LEC NPs in vitro, but also increase the inhibitory function of PCPs on NSCLC cells. To further verify these effects, cell migration analysis of H1299, A549 and LETP-a2 cells, after treatment by digestive PCPs, PCPs@LEC–CS or PCPs@LEC–CS–PEG for 24 h and 48 h, was employed (Figure 8). The corresponding cell images are presented in Supplementary Figures S1–S3. It is worth mentioning that the exposure of digestive PCPs (PCP1-3) does not significantly inhibit the migration of LETP-a2 cells, suggesting that the active peptides might be destroyed during the simulated gastric fluid (SGF) digestion. Strikingly, the migration of NSCLC cells significantly decreased after SGF-PCPs@LEC–CS (PCP1: 22.21% ± 2.45%, PCP2: 17.89% ± 3.23%, PCP3: 14.67% ± 1.24%) and SGF-PCPs@LEC–CS–PEG (PCP1: 23.66% ± 3.13%, PCP2: 21.09% ± 4.23%, PCP3: 19.67% ± 5.01%) with PCPs@LEC–CS (PCP1: 26.03% ± 2.15%, PCP2: 25.47% ± 3.21%, PCP3: 24.69% ± 1.04%), the PEG modified liposomes showed a more obvious inhibitory effect on cell migration in 48 h of treatment. Taken together, these results have indicated that PCPs@LEC–CS–PEG exerts good antineoplastic and anti-digestive functions, and could be used as a potential functional peptide liposome complex.

## 4. Discussion

In this work, the stabilization and function of phycocyanin-derived peptides loaded by nanoparticles (NPs) were investigated. The embedding of LEC liposomes could significantly increase the stability of PCPs through promoting their resistance to high temperature (68.256% ± 3.26%), pH (60.17% ± 3.67%) and protease. Modification of NPs by PEG and CS could enhance the protective effects on PCPs. Furthermore, in vitro phenotypic experiments confirmed that the inclusion of PCPs@PEG-CS–LEC NPs also significantly increased the inhibitory activities of PCPs against multiple NSCLC cells including A549, H1299 and LTEP-a2 cells, compared with non-embedded PCPs.

Bioactive peptides have shown that they can have different interactions in the body that modulate physiological processes in different metabolic pathways in the organism, for instance, inhibiting the action of certain enzymes [30]. Actually, bioactive peptides from food sources have been found to exert various physiological functions including anticancer [31,32,33]. There have been some reports on the identification and functional studies of bioactive peptides derived from phycocyanin, most of which are related to improving metabolic or inflammatory diseases. For instance, Liu and Li et al. both reported the therapeutic effect of phycocyanin peptides on pulmonary disease in vivo and in vitro [6,22]. The alleviative effect of phycocyanin peptides against inflammatory bowel disease (IBD) was also investigated by Xu et al. through the zebrafish model [34].

In fact, although the antineoplastic activity of phycocyanin has been widely acknowledged [35,36], studies on the identification and modification of anticancer peptides of phycocyanin are rarely reported. Particularly, Liu et al. have investigated the inhibitory effects of a phycocyanin-derived peptide on pulmonary fibrosis based on an A549 cell model [6], but they did not conduct functional evaluations on individual peptide segments. Previously, three peptides (PCP1: AGDASVLEDR, PCP2: ADSLLSGLR and PCP3: MFDAFTK) derived from phycocyanin with NSCLC cell inhibition effects were successfully identified in our investigation [8].

In this study, LEC and CS were utilized to encapsulate three peptides (PCP1-3) in liposomes, and the encapsulation efficiency reached over 70%, indicating that the combination of LEC and CS could effectively protect PCPs. Interestingly, under the same processing conditions, the maximum embedding rate of PCP1 was approximately 92%, that of PCP2 was approximately 80%, and that of PCP3 was approximately 73%. It is worth noting that although PCP1 has a larger molecular weight among the three peptides, the abundant basic and hydrophobic amino acids (R, A, V, L) it contains might facilitate its binding with lecithin, thereby enhancing the encapsulation efficiency of the liposomes for peptide segmentation [37]. Nevertheless, the addition of PEG significantly enhanced the encapsulation efficiency of PCP1-3, which laid the foundation for the subsequent experiments.

The bioactive sequences must be maintained after digestion to reach the organ where they have to perform their function [38]. Like proteins, the stability of peptides is easily affected by factors such as temperature, thus weakening their physiological activities. One strategy to overcome the limitation of the poor stability and bioavailability of peptides, given their propensity to be degraded during preservation and digestion, is their encapsulation [39]. It is worth noting that Yang et al. have already explored the embedding of phycocyanin using chitosan, creating C-phycocyanin/carboxymethyl chitosan-CD59-specific ligand peptide nanoparticles, which could be targeted and delivered to Hela cells [40]. Yang’s research has provided ideas for the targeted delivery of functional proteins. Although the focus of this study was not on targeted delivery, we improved the encapsulation technology of PCPs and adopted the combined encapsulation of LEC/CS/PEG, which could maximize the protective effect on phycocyanin peptides. Compared with the encapsulation rate of Yang’s research (65%), the encapsulation rate of PCPs in this work has increased by over 10% (75–80%).

In this study, liposomes with LEC as the main skeleton were selected for protecting and embedding PCPs. Different from traditional carriers, the modification of LEC liposomes by CS and PEG-2000 seems able to enhance the stability of NPs. According to the current results, LEC–CS–PEG NPs have a stronger protective effect on PCPs than LEC liposomes alone, which is mainly reflected in the resistance of NPs to high temperature and protease digestion in vitro. Subjecting food to high temperatures could modify proteins and peptides, leading to denaturation or aggregation [41]. Interestingly, some studies show that heat treatments do not significantly affect the bioactivity of peptides, such as the ACE inhibitory activity of ham peptides after high temperature (117 °C) for prolonged times (up to 60 min) [42], which might be due to their specific sequences and structures. In contrast, functional peptides derived from phycocyanin are highly sensitive to temperature. The current work has confirmed that CS-PEG modifications could increase the NPs’ ζ-potential and improve their embedding efficiency of PCPs, providing a certain theoretical basis for the in-depth utilization of PCPs in the food industry or the drug delivery field.

In addition, the inclusion of PCPs by LEC–CS–PEG NPs greatly improves its anti-digestive ability in vitro, and is closely related to the antineoplastic activity of PCPs. According to the current results, the survival rate of NSCLC cells increased after protease treatment, indicating that digestion could weaken the anticancer activity of NPs to a certain extent. However, compared with the unprotected PCPs, its antineoplastic function is significantly improved. This indicates that the protection provided by liposomes can enhance the resistance of PCPs to extreme external environments. Due to the protective effect of substances like PEG, large molecules like proteins and enzymes find it difficult to damage the intact structure of the liposomes, thereby providing protection for PCPs. As for the mechanism, in fact, our previous research has reported that phycocyanin protein could inhibit the functions of NSCLC cells through regulating the activity of the AKT and NF-kB signaling pathways [43,44], which might be a possible mechanism by which PCPs regulate the activity of NSCLC cells. The outcome of the present study could provide a scientific basis for applications including the targeted transportation of functional substances, and the efficient preservation and utilization of functional foods.

However, although this study investigated the preparation and characterization of liposomes, as well as the effect of encapsulating PCPs on cell viability, the function and regulatory mechanism of PCPs in vivo still require further exploration and investigation. Nevertheless, the results of this study have confirmed the protective effect of LEC/CS/PEG on PCP encapsulation, which can lay an important foundation for subsequent in-depth research.

## Data Availability

The data that support the findings of this study are available from the corresponding author upon reasonable request.

## Supplementary Materials

The following supporting information can be downloaded at: https://www.mdpi.com/article/10.3390/foods14203487/s1, Figure S1: Cell migration analysis of digestive PCP1@LEC-CS-PEG NPs on NSCLC cell lines including A549, H1299 and LTEP-a2 cells.; Figure S2: Cell migration analysis of digestive PCP2@LEC-CS-PEG NPs on NSCLC cell lines including A549, H1299 and LTEP-a2 cells; Figure S3: Cell migration analysis of digestive PCP3@LEC-CS-PEG NPs on NSCLC cell lines including A549, H1299 and LTEP-a2 cells; Table S1: The thermal stability analysis of PCPs@LEC-CS-PEG NPs; Table S2: The pH stability analysis of PCPs@LEC-CS-PEG NPs; Table S3: The storage stability analysis of PCPs@LEC-CS-PEG NPs.

## Abbreviations

The following abbreviations are used in this manuscript:

NSCLC	Non-small cell lung cancer
PCPs	Phycocyanin-derived peptides
NPs	Nanoparticles
CS	Chitosan
LEC	Lecithin
PEG	Polyethylene glycol
FTIR	Fourier transform infrared
IBD	Inflammatory bowel disease
ACE	Angiotensin I converting enzyme
PC	Phycocyanin
