# Supplementary material for: Preparation, Stability and In Vitro Antineoplastic Function of Lecithin–Chitosan–Polyethylene Glycol Nanoparticles Loaded with Bioactive Peptides Derived from Phycocyanin
Source: Foods. 2025 Oct 13;14(20):3487. doi: 10.3390/foods14203487 (PMC12563087; doi:10.3390/foods14203487)
Supplement: Supplementary file 1 [file foods-14-03487-s001.zip › Supplementary Figures.pdf]

Supplementary Figures

Figure S1

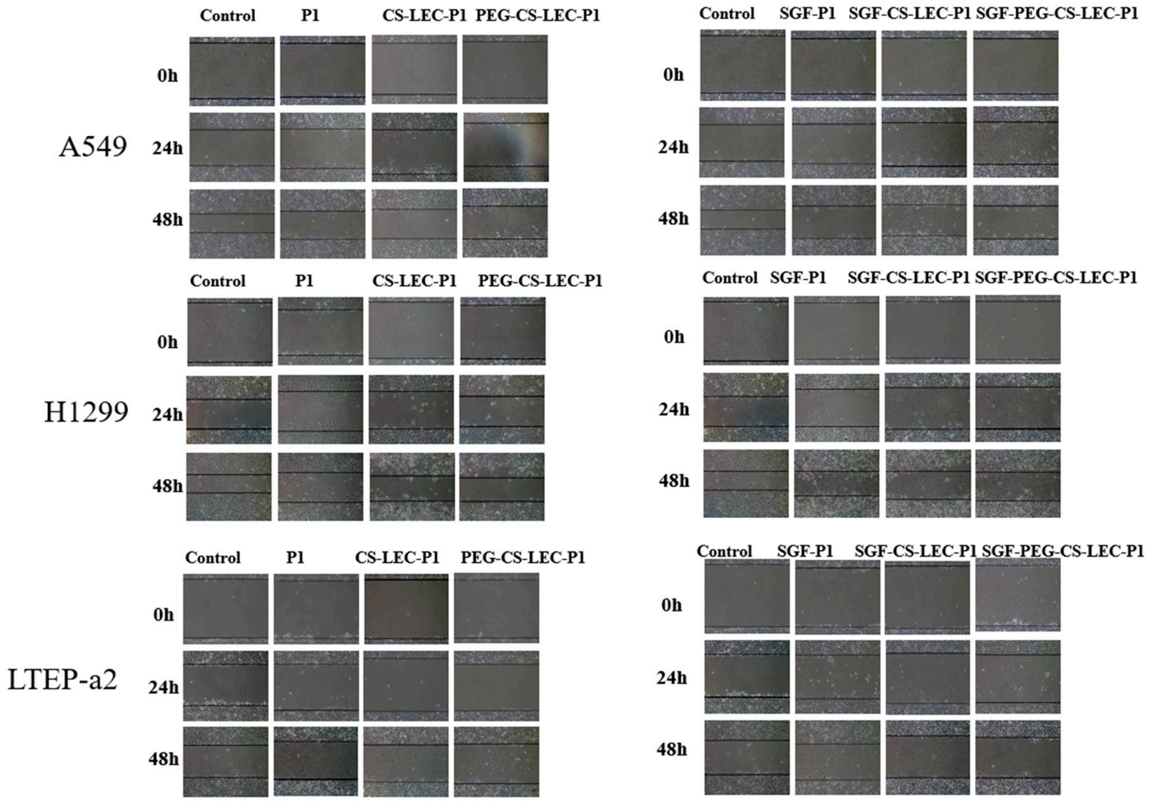

Figure S1. Cell migration analysis of digestive PCP1@LEC-CS-PEG NPs on NSCLC cell lines including A549, H1299 and LTEP-a2 cells.

Figure S2

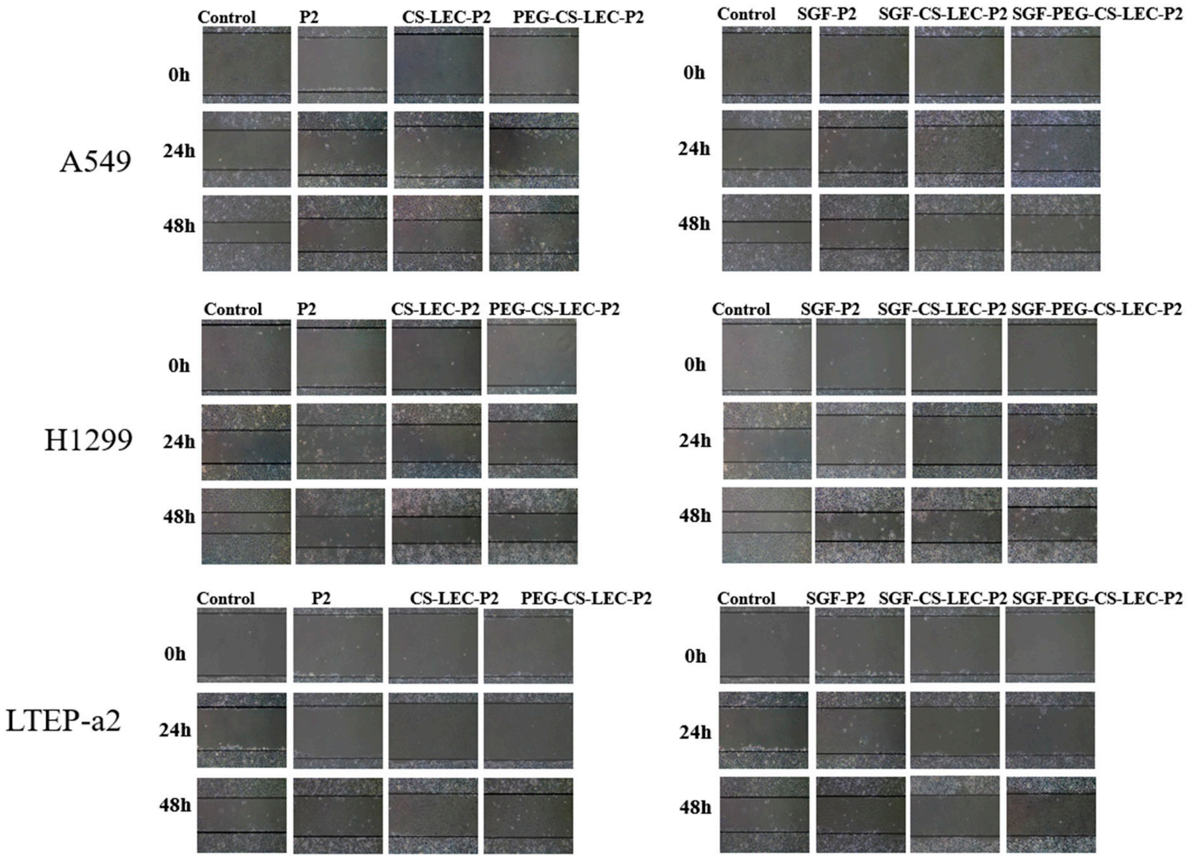

Figure S2. Cell migration analysis of digestive PCP2@LEC-CS-PEG NPs on NSCLC cell lines including A549, H1299 and LTEP-a2 cells.

Figure S3

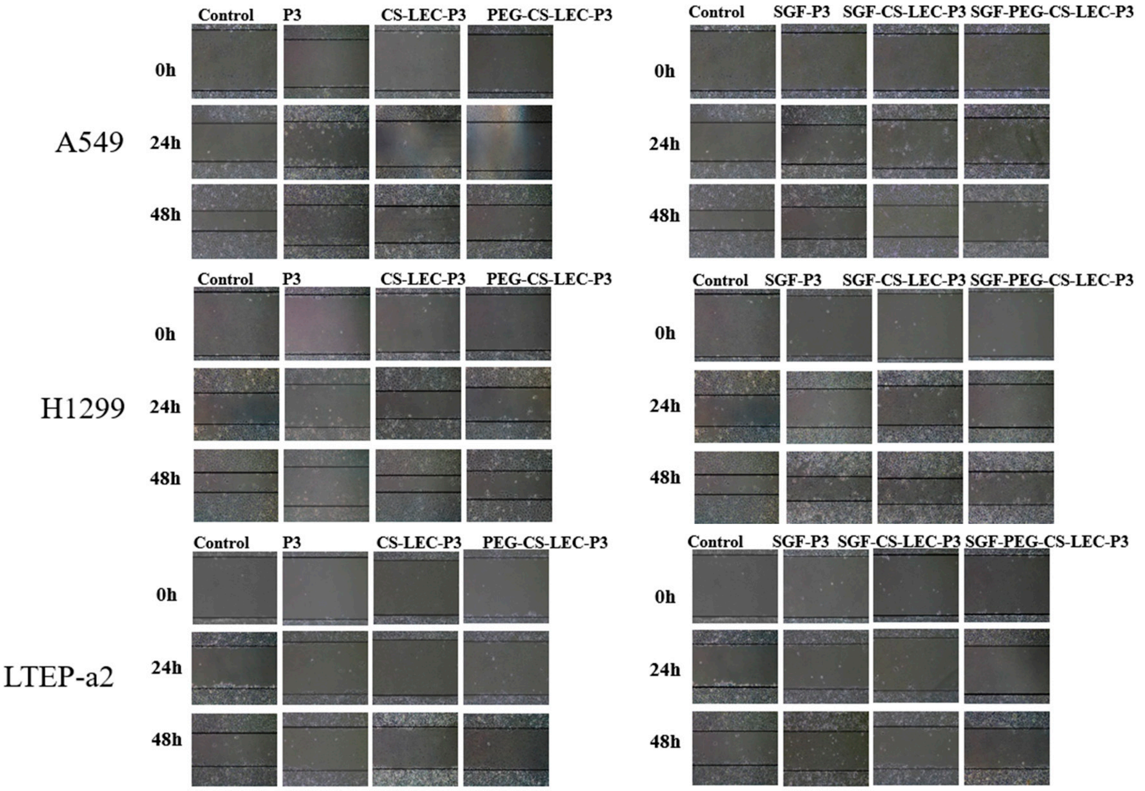

Figure S3. Cell migration analysis of digestive PCP3@LEC-CS-PEG NPs on NSCLC cell lines including A549, H1299 and LTEP-a2 cells.
