# Supplementary material for: Preparation, Stability and In Vitro Antineoplastic Function of Lecithin–Chitosan–Polyethylene Glycol Nanoparticles Loaded with Bioactive Peptides Derived from Phycocyanin
Source: Foods. 2025 Oct 13;14(20):3487. doi: 10.3390/foods14203487 (PMC12563087; doi:10.3390/foods14203487)
Supplement: Supplementary file 1 [file foods-14-03487-s001.zip › Supplementary Tables.pdf]

Table S1. The thermal stability analysis of PCPs@LEC-CS-PEG NPs

Sample	T(80 ºC)	Dh(nm)	PDI	EE(%)	Sample	T(80 ºC)	Dh(nm)	PDI	EE(%)	Sample	T(80 ºC)	Dh(nm)	PDI	EE(%)
PCP1@LEC	0 min	68.52 ± 0.05a	0.22 ± 0.02c	46.26 ± 0.26a	PCP2@LEC	0 min	62.56 ± 0.27b	0.27 ± 0.03b	59.16 ± 1.26a	PCP3@LEC	0 min	65.27 ± 0.28a	0.30 ± 0.01a	40.33 ± 2.15a
	20 min	65.26 ± 0.59a	0.31 ± 0.01a	40.41 ± 2.13a		20 min	68.29 ± 0.59a	0.31 ± 0.02a	38.64 ± 1.57b		20 min	68.26 ± 0.15a	0.26 ± 0.03b	38.23 ± 1.60a
	40 min	68.06 ± 0.96a	0.21 ± 0.03c	36.26 ± 1.22a		40 min	62.18± 0.46b	0.30 ± 0.02a	36.11 ± 2.56b		40 min	70.26 ± 2.14a	0.30 ± 0.05a	40.26 ± 2.14a
	60 min	63.26 ± 1.25a	0.26 ± 0.013b	31.17 ± 2.66a		60 min	68.27 ± 1.24a	0.27 ± 0.02b	31.29 ± 1.95c		60 min	64.15 ± 1.25a	0.31 ± 0.10a	31.26 ± 2.15b
PCP1@LEC-CS	0 min	100.26 ± 2.16b	0.23 ± 0.03a	59.26 ± 3.26a	PCP2@LEC-CS	0 min	105.95 ± 2.16a	0.20 ± 0.02b	75.27 ± 2.15a	PCP3@LEC-CS	0 min	115.27 ± 2.16a	0.20 ± 0.01b	52.17 ± 4.16a
	20 min	96.16 ± 3.25b	0.22 ± 0.02b	55.27 ± 2.47a		20 min	112.26 ± 0.96a	0.19 ± 0.02a	74.26 ± 1.52a		20 min	109.26 ± 1.25a	0.20 ± 0.01a	50.76 ± 2.56a
	40 min	106.16 ± 2.16a	0.17 ± 0.01a	53.62 ± 1.25a		40 min	116.75 ± 1.26a	0.21 ± 0.02a	72.26 ± 2.16a		40 min	108.45 ± 3.25a	0.23 ± 0.02b	48.01 ± 3.16a
	60 min	110.23 ± 0.26a	0.21 ± 0.02c	53.26 ± 4.26a		60 min	108.22 ± 1.25a	0.20 ± 0.01b	73.16 ± 4.25a		60 min	112.56 ± 1.95a	0.19 ± 0.02b	44.35 ± 3.56a
PCP1@LEC-CS-PEG	0 min	130.25 ± 0.96a	0.15 ± 0.02c	74.17 ± 2.17a	PCP2@LEC-CS-PEG	0 min	128.97 ± 0.67a	0.21 ± 0.02b	79.16 ± 3.16a	PCP3@LEC-CS-PEG	0 min	135.26 ± 3.16a	0.16 ± 0.02b	58.16 ± 2.17a
	20 min	126.36 ± 1.24a	0.25 ± 0.03a	71.11 ± 3.27a		20 min	130.16 ± 1.26a	0.22 ± 0.03a	74.56 ± 2.65b		20 min	132.15 ± 2.15a	0.20 ± 0.02a	55.15 ± 3.08a
	40 min	124.06 ± 0.65a	0.20 ± 0.02b	70.16 ± 2.16a		40 min	129.24 ± 2.15a	0.20 ± 0.02b	74.26 ± 1.69b		40 min	128.26 ± 3.21a	0.16 ± 0.03b	55.26 ± 2.36a
	60 min	132.23 ± 1.25a	0.23 ± 0.02a	68.26 ± 3.26a		60 min	118.33 ± 0.65b	0.20 ± 0.01b	72.16 ± 3.26b		60 min	134.16 ± 0.95a	0.20 ± 0.02a	53.56 ± 3.54a

Table S2. The pH stability analysis of PCPs@LEC-CS-PEG NPs

Sample	pH	Dh(nm)	PDI	EE(%)	Sample	pH	Dh(nm)	PDI	EE(%)	Sample	pH	Dh(nm)	PDI	EE(%)
PCP1@LEC	2	65.36±0.02c	0.21±0.05b	40.00±1.28a	PCP2@LEC	2	60.76±1.25b	0.25±0.03c	30.26±2.15b	PCP3@LEC	2	68.17±0.27c	0.32±0.02a	45.27±0.99a
	4	68.52±0.05c	0.22±0.02b	33.26±2.12b		4	62.26±0.27b	0.17±0.03c	39.16±1.26a		4	65.93±0.27c	0.30±0.01b	40.33±2.16b
	6	75.16±0.29b	0.22±0.03b	30.71±1.26b		6	65.92±0.65b	0.36±0.01a	26.54±2.16b		6	75.66±0.87b	0.32±0.02a	35.62±1.95c
	8	89.23±1.25a	0.36±0.03a	28.18±0.66b		8	70.84±0.89a	0.30±0.03b	19.56±4.25c		8	80.26±1.26a	0.29±0.15b	33.29±2.67c
PCP1@LEC-CS	2	99.26±0.66c	0.77±.012c	55.76±2.16a	PCP2@LEC-CS	2	112.16±3.25c	0.30±0.03c	45.27±1.25b	PCP3@LEC-CS	2	124.26±1.25c	0.26±0.016c	60.26±2.15a
	4	100.17±2.16c	0.23±0.03c	50.43±3.25a		4	105.95±2.16c	0.20±0.02d	49.26±2.16b		4	115.37±2.16c	0.20±0.01c	52.17±4.11b
	6	325.54±5.27b	0.52±0.03b	48.26±1.56a		6	301.54±1.26b	0.53±0.02b	36.77±0.18a		6	398.57±3.16b	0.60±0.02b	46.46±4.22c
	8	869.52±4.26a	0.85±0.13a	33.65±2.18a		8	1009.27±6.06a	0.95±0.10a	25.41±5.15a		8	945.66±2.12a	1.00±0.09a	40.26±2.68d
PCP1@LEC-CS-PEG	2	126.26±0.66b	0.15±0.03b	58.17±1.56a	PCP2@LEC-CS-PEG	2	135.23±0.59c	0.20±0.03c	52.16±2.16a	PCP3@LEC-CS-PEG	2	130.36±2.15c	0.26±0.01c	62.16±3.28a
	4	130.65±0.96b	0.16±0.02b	55.13±2.17a		4	128.27±0.66c	0.21±0.02c	55.16±3.16a		4	135.26±3.16c	0.16±0.02d	58.16±2.11b
	6	126.17±1.23b	0.31±0.02a	50.26±3.19b		6	159.25±0.28b	0.30±0.03b	51.72±1.28a		6	205.37±3.26b	0.35±0.07b	51.82±1.16c
	8	236.44±6.22a	0.35±0.09a	48.82±3.12b		8	325.56±5.18a	0.43±0.03a	42.44±3.12b		8	456.57±10.29a	0.43±0.04a	42.54±5.11c

Table S3. The storage stability analysis of PCPs@LEC-CS-PEG NPs

Sample	Days	Dh(nm)	PDI	Sample	pH	Dh(nm)	PDI	Sample	pH	Dh(nm)	PDI
PCP1@ LEC	0	62.74±1.26b	0.36±0.02c	PCP2@ LEC	0	68.76±0.95b	0.37±0.02c	PCP3@ LEC	0	66.49±0.96d	0.34±0.02c
	7	156.40±2.01a	0.40±0.01b		7	162.16±2.15a	0.33±0.03d		7	126.25±1.26c	0.32±0.02c
	14	151.00±2.27a	0.42±0.02a		14	168.20±2.02a	0.40±0.01b		14	142.50±3.25b	0.37±0.02b
	21	157.50±3.16a	0.44±0.01a		21	166.24±1.26a	0.42±0.05a		21	162.13±2.59a	0.41±0.02a
PCP1@ LEC-CS	0	149.20±2.65c	0.11±0.02b	PCP2@ LEC-CS	0	132.20±3.26d	0.16±0.01b	PCP3@ LEC-CS	0	136.21±6.26d	0.12±0.01c
	7	177.60±3.13b	0.20±0.03a		7	143.64±4.24c	0.17±0.03b		7	144.19±5.16c	0.16±0.01b
	14	185.11±2.66a	0.21±0.01a		14	169.11±5.22b	0.20±0.03a		14	177.54±4.22b	0.19±0.02a
	21	188.74±4.26a	0.22±0.01a		21	179.27±2.11a	0.21±0.05a		21	189.27±3.22a	0.20±0.02a
PCP1@ LEC-CS-PEG	0	131.30±2.37c	0.18±0.01b	PCP2@ LEC-CS-PEG	0	136.20±3.27c	0.16±0.01c	PCP3@ LEC-CS-PEG	0	126.93±1.60d	0.17±0.01c
	7	166.91±8.59b	0.18±0.01b		7	133.51±3.15c	0.20±0.01b		7	142.22±2.56c	0.20±0.01b
	14	193.36±6.16a	0.20±0.01a		14	179.63±4.24b	0.19±0.02b		14	168.32±3.26b	0.19±0.02b
	21	199.84±4.59a	0.23±0.01a		21	185.43±3.15a	0.23±0.01a		21	196.35±4.22a	0.23±0.01a
